# G-quadruplexes in the proximity 3′-UTR enhances alternative polyadenylation of Neogenin 1

**DOI:** 10.1016/j.isci.2025.113952

**Published:** 2025-11-10

**Authors:** Pauline Lejault, François Bolduc, Marc-Antoine Turcotte, Jean Pierre Perreault

**Affiliations:** 1 Université de Sherbrooke, Sherbrooke, QC J1E 4K8, Canada; 2 Department of Biochemistry and Functional Genomic, Pavillon de recherche appliqué sur le cancer, Université de Sherbrooke, Sherbrooke, QC J1E 4K8, Canada

**Keywords:** molecular biology, molecular mechanism of gene regulation, molecular interaction, molecular network

## Abstract

Polyadenylation is a crucial step in mRNA maturation. Fifty percent of the time alternative polyadenylation sites (PASs) are used instead of canonical ones, affecting the length of the transcripts’ 3′-UTRs. The mechanisms controlling the selection of these alternative PASs remain unknown. Using a unique sequencing method (PolyA Click-seq) to identify different polyadenylated isoforms, PolyA events modulated by RHPS4, a ligand known to stabilize RNA G-quadruplex structures (rG4s), were revealed. Through *in silico* selection, *in vitro* assays, and rG4 mutagenesis, the pivotal role of rG4 structures in determining PASs was uncovered, most notably in the case of the gene encoding Neogenin-1 (NEO1), a cell surface protein that is a member of the immunoglobulin superfamily. Our findings highlight the importance of rG4-mediated alternative polyadenylation (APA) regulation in the 3′-UTR as a method to both alter isoform choice and impact protein synthesis, with potential relevance for RNA-based therapeutics.

## Highlights

• PolyA Click-seq reveals G4 enrichment at alternative polyadenylation sites
• RHPS4 reshapes global mRNA polyadenylation site usage
• G4 structures in 3′-UTRs drive alternative polyadenylation of NEO1
• G4-mediated regulation alters 3′-UTR length

## Graphical abstract

**Figure:**
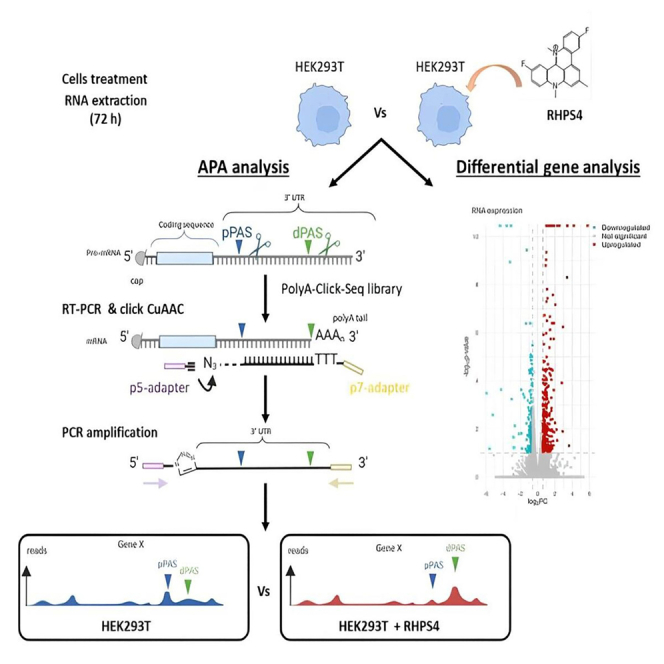

## Introduction

Advances in RNA biology have enabled a range of RNA-based therapeutics, including mRNA vaccines and antisense oligonucleotides.^1^ The development of therapeutics requires a deeper understanding of RNA structures and their interactions with host molecules in cells, particularly how mRNA stability can be modulated to induce a prolonged and more efficient expression and response. In eukaryotes, pre-messenger RNA (pre-mRNA) maturation requires both splicing and polyadenylation. Polyadenylation, the addition of a polyadenine tail at the 3′ end of pre-mRNA, is essential for mRNA export, stability, and translation.^2^ Numerous pre-mRNAs contain multiple polyadenylation sites (PASs) which yield alternative isoforms with different PolyA tails. This phenomenon, known as alternative polyadenylation (APA), is widespread, affecting 50%–70% of transcripts, and significantly shapes both the transcriptome (through 3′-UTR length) and the proteome (via altered coding sequences). It impacts not only RNA and protein levels but also subcellular localization and even protein functions.^3^^,^^4^ Unlike splicing, APA site recognition proceeds sequentially and appears to be independent of whether it is the distal (i.e., that with a long 3′-UTR) or the proximal isoform (i.e., that with a short 3′-UTR). While deregulation of APA has been linked to numerous pathological conditions such as cancer, neuropathologies, and cardiac deregulation, the precise mechanisms driving these dysfunctions remain elusive.^5^

RNA G-quadruplex structures (rG4s) are RNA guanosine-rich sequences that fold into non-canonical 4-stranded helical structures. Depending on their localization, rG4s are suspected to fine-tune both the pre-mRNA and the mature mRNA lifecycles. For instance, when located in the 5′-UTR they affect the accessibility of the mRNA to the translation machinery, resulting in either the promotion or the inhibition of translation.^6^^,^^7^ In pre-mRNA, they can modulate alternative splicing events by acting as splicing regulatory elements, thus impacting either the inclusion or the exclusion of specific exons in the mature mRNA.^8^ The involvement in APA of rG4 located in the 3′-UTRs of two transcripts (LRP5 and FXR1) was unexpectedly discovered in 2013.^9^ More precisely, it was found that rG4 structures located 49 and 60 nts from APA sites in LRP5 and FXR1, respectively, could influence the selection of APA sites, leading to shorter, alternative transcripts. Additionally, it was found that this could interfere with the miRNA regulatory networks.^9^ Despite the critical importance of APA in both pre-mRNA stability and functional protein production, the formation of secondary structures and their implications therein remain largely unknown.

In this study, the role of rG4s in APA at the transcriptome level was explored using the innovative PolyA Click-seq methodology. Strikingly, these analyses revealed that the small molecular tool RHPS4, which is known to be a G4-ligand, induced significant alterations in the PolyA profiles within cells. Among these alterations, an investigation into the Neogenin-1 transcript, a gene that is linked to cancer,^10^^,^^11^^,^^12^ revealed that the formation of the distal 3′-UTR rG4 seemed to exert a stronger influence on both the selection of the PAS and on the production of long isoforms than did the formation of the proximal 3′-UTR rG4. These findings carry significant implications for both mRNA maturation and protein production. Previous research, including our own, has shown that therapeutic targeting of the 3′-UTR can alter protein production.^5^^,^^9^ Here, a layer of APA regulation involving rG4 is unveiled. The data suggest that targeting rG4 mediated APA regulation could hold promise as a therapeutic strategy for several previously mentioned disorders, especially cancer.^10^^,^^11^^,^^13^

## Results

### A dataset of APA is induced by RHPS4

**Figure 1:**
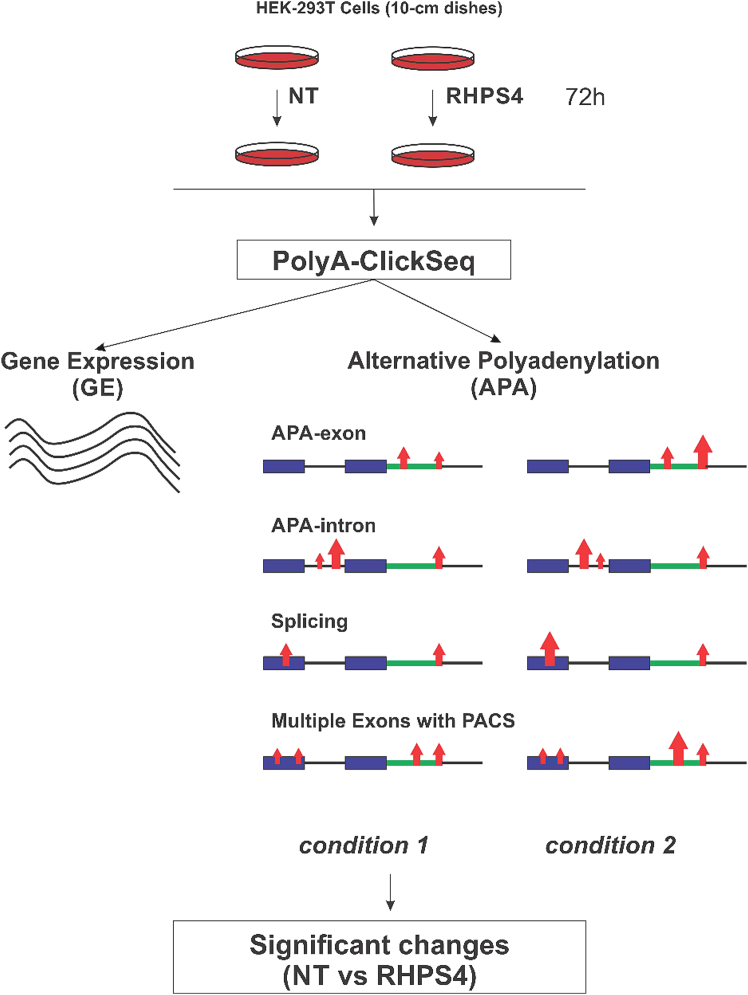
Exploring RHPS4-induced alterations in APA events—A comprehensive guide in HEK-293T cells Schematic overview of PolyA Click seq (PAC-seq). After several key steps, including RT-PCR, Click-chemistry, and PCR amplification, the final library consists of DNA fragments containing the Illumina p5 adaptor, a portion of the UTR, a stretch of adenine derived from both the RNA template and the poly(T) primer and, finally, the p7 Illumina indexing primer. Following PAC-seq, the use of DESeq2 analysis permitted the determination of the mRNA expression levels, while the DPAC software analysis permitted the differentiation of the various APA events such as APA-exon, APA-intron, splicing, and multiple exons with PACS.

In order to highlight the role of potential G4s (pG4) as universal regulators of APA, a well-established G4-ligand, 3,11-Difluoro-] acridinium methylsulfate (RHPS4), was used in HEK-293T cells.^14^ RHPS4 had previously been identified as a potent inducer of alterations in the RNA G4 landscape across both coding and non-coding mRNAs, leading to the anticipation of modifications in the APA landscape.^15^^,^^16^^,^^17^ Additionally, this G4 ligand has been described as having no G4-inducer effect on single guanine-rich RNA strands, unlike other well-known G4 ligands. Using RHPS4 also takes advantage of working with a more authentic population of G4s.^17^ In order to determine the ideal RHPS4 concentration for use in HEK-293T cells, the inhibitory concentration of RHPS4 was determined by MTT assay (Figure S1). Based on these results, the cells were treated at the IC_20_ (1.5 μM) of RHPS4 for 72 h. Following treatment, total RNA was isolated and libraries were prepared so as to be able to conduct PolyA click-seq (-PAC-Seq) aimed at identifying both significant alterations at the APA level and modifications in gene expression (Figure 1).^18^^,^^19^ Among all the specific 3′-UTR sequencing technologies described (e.g., 3′ READs^+^, FLAM-seq, and PAIso-Seq),^20^^,^^21^^,^^22^ this elegant approach has been chosen due to its comprehensive view of the mRNA and its correlation with the search for G4. These innovative approaches included both the use of both computational tools, in order to be able to re-analyze the RNA-seq data and identify APA events, and of customized targeted sequencing approaches using click-chemistry that focused on the 3′-end of the mRNA. A described previously,^18^^,^^19^ the raw data was analyzed using Differential PolyA-Clustering (DPAC) for the preprocessing of the polyA-tail targeted RNAseq data. This pipeline includes the mapping of the polyA sites, clustering and annotating them, and determining the usage of differential polyA-clusters (located upstream of the PAS) through DESeq2.^23^ The raw data were also used to simultaneously measure changes in gene expression, as well as for detecting APA events with high confidence, comparing treated and non-treated cells in triplicate (accessible online).The use of RHPS4 does not exclude the discovery of new PACs, although these are not frequent in human cells. The DPAC allows for their detection if they are located within an annotated gene (exon/intron).

The conclusive omics analysis of 14,819 genes obtained from PAC-Seq resulted in the identification of four major changes caused by the addition of RHPS4.

**Figure 2:**
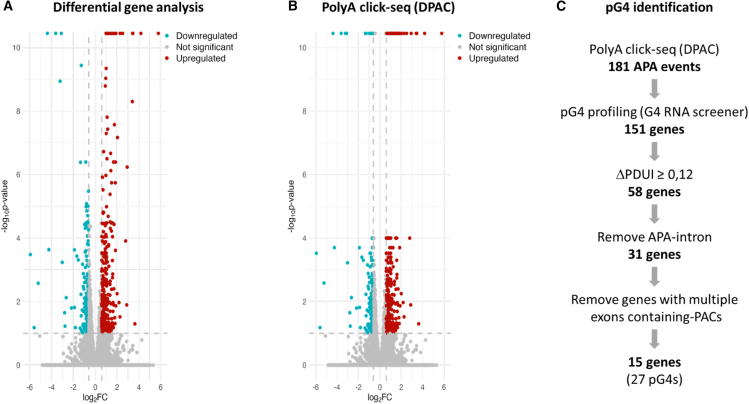
Characterizing the changes induced by RHPS4 treatment—Impact on both gene expression and polyadenylation site usage (A) Output visualization of the DESeq2 results with a Log2 fold change versus the *p* adjusted value for the genes detected in PAC seq. The genes with a fold change of greater than 1.5 and an adjusted *p* value of less than 0.1 are colored in red for the upregulated genes and in blue for the downregulated genes. (B) DPAC results: any APA changes located at the 3′-UTR ends with a magnitude exceeding 10% and a *p* value of <0.1 are being shown. Significant downregulation is indicated in blue, while significant upregulation is indicated in red. (C) Bioinformatic pipeline for rG4 detection. Starting with the 181 genes initially identified (DPAC with significant variation = lengthening, shortening, or both), a search using G4 RNA Screener for the presence of potential rG4s was carried out both 100 nt upstream and downstream of the genomic position of the identified PACs. Subsequently, only DPDUIs greater than 0.12 were retained. All situations containing more than one exon, or involving an intron, were also removed.

**Figure 3:**
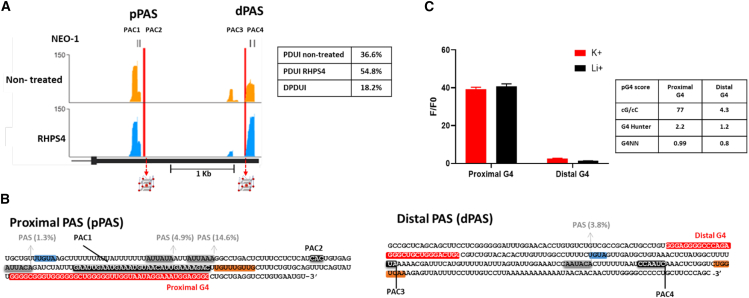
*In vitro* analysis of the potential G4 sequences found in the area surrounding both the proximal and distal polyA sites (A) Genomic view of the mapped reads found in non-treated and in RHPS4-treated HEK-293T cells in the NEO1 3′-UTR. The PACs are identified by bars located in the upper part of the graph (and are numbered 1–4). The red vertical lines represent the position of the pG4s. On the right, a table with the PDUIs obtained for NEO1 from DPAC for each condition is presented. (B) The sequence details of both the proximal and the distal PASs are shown as follows: the *cis*-elements are highlighted; the polyA signals sites and their percentages of use are shaded in gray 35; the PAC sites are marked in black; the UGUA motif is indicated in blue; the G/G-U-rich elements (called DSE elements) are highlighted in orange; and, the potential G-quadruplex region is depicted in red. (C) Fluorescence assays were performed on *in vitro* transcripts of pG4s derived from both the proximal and distal regions of the NEO1 3′-UTR, under conditions with either K+ or Li+ being present. The histogram illustrates the fluorescence ratio of the G4-light-up fluorophore (NMM) in the presence of RNA compared to that of NMM alone (F/F0). The analysis also includes the pG4 formation, which integrates the three scores derived from the G4 RNA screener.
(2) Significant changes in the APA: as expected, treatment with the RHPS4 ligand caused several changes in the APA landscape (Figure 2B). A total of 297 significant changes were identified, including both up- and downregulated events (Table S1). These changes were characterized by the differential usage pattern of a single PAC as indicated by an independent hypothesis weighting (IHW) *p* adjust of <0.1, resulting in a fractional change of PAC usage of at least 10%. While this threshold is not overly stringent, it represents a balanced and commonly accepted cutoff consistent with DESeq2 default settings that is well-suited to capturing biologically relevant changes without inflating false positives in this type of transcriptome-wide analysis.
(3) Changes in the localization of each APA event (i.e., APA-exon, APA-intron, splicing, and multiple exons with PACS^2^^,^^23^^,^^24^^,^^25^): the relative locations of PACs determine whether APA results in 3′-UTR shortening or lengthening. If two PACs (each with ≥5% occupancy) are within a single exon, a change in the usage of one can led to either the APA-induced shortening or lengthening of the 3′-UTR. If three or more PACs are found and one or both of the middle PACs changes in abundance, this can affect upstream and downstream PACs, resulting in both shortening and lengthening phenotypes, annotated as “both.” And in the complex case of a splicing event, two PACs (or more) are present, located in different exons or introns, affecting both the length and the constitutive splicing. Among the APA events induced by RHPS4, 86 events were classified as “splicing,” 36 as “shortening,” 86 as “lengthening,” and 59 as having a dual effect, termed “both” (Tables S3 and S4).
(4) Changes in the quantitative point of view of each APA event: 3′-UTR length changes can be quantified using the Percentage of distal PolyA site usage index (PDUI). Differences in APA usage between normal and treated cells identify both lengthening (positive index) and shortening (negative index) of 3′-UTR (Table S1). Out of the 27,548 exons in which a PAC was detected (in a total of 14,819 genes), 37% exhibited a change in PDUI per exon following RHPS4 treatment. All raw data are available online (see STAR Methods).

(1) Changes in the global RNA differential expression levels: out of 14,819 analyzed genes, 498 genes exhibited a significant variation in their expression levels (>1.5-fold and having a *p* adjust of <0.1), were identified. A significant upregulation variation was noted for 304 genes, and a similar downregulation variation was noted for 194 genes when RHPS4-treated cells were compared with untreated control cells (Figure 2A). The lists of both the up- and downregulated genes, along with their corresponding Gene Ontology (GO) annotations, are presented in Table S2 and Figures 2 and 3. The top upregulated genes are involved in the stress response and in the regulation of apoptosis signaling.

The widespread presence of G4 structures in nucleic acids suggests that treatment with G4-ligands such as RHPS4 can modulate RNA expression, as has been demonstrated in previous studies with MCF7 cells.^15^ Here, the above findings demonstrate that the use of a G4-ligand can induce notable changes in the APA profile of HEK293T cells are presented which could have significant impacts on mRNA stability, translation efficiency, nuclear export, cellular localization, and the localization of the encoded protein. The global impact of G4 stabilization using RHPS4 underscores the potential significance of G4 structures in regulating APA processes.

### Potential G4 forming sequences are found near PACs with APA

In the entire transcriptome, more than 1 million pG4 sequences are suspected to modulate a plethora of mechanisms, including translation, splicing and polyadenylation.^26^ In this study, rG4s involved in APA were selected in order to be able to conduct more specific *in vitro* experiments. To do so, a search near the PACs’ genomic positions (i.e., within 100 nt both upstream and downstream of the PAC) was conducted in order to be able to identify the presence of these structures using a bioinformatics tool, G4 RNA screener.^27^ As the use of RHPS4 is known to have a global impact on G4 formation, the goal here was to highlight the role of rG4 structures in the APA changes induced by RHPS4. The G4 RNA screener is designed to identify regions in the transcriptome with a probability of forming pG4s. G4 RNA screener integrates three scoring systems: the consecutive G over consecutive C score (cG/cC), the G4Hunter score (G4H), and the G4 neuronal network score (G4NN). The entire bioinformatics pipeline is available online (see STAR Methods). For this study, sequences were considered positive if they obtained at least one score out of the three being higher than the following thresholds: G4Hunter > 0.9, G4NN > 0.5, and cG/cC > 4.5. In order to specifically investigate APA events influencing mRNA length modulation, genes annotated with “splicing” were excluded, and the analysis was focused on other events related to “shortening,” “lengthening,” and “both.” This resulted in a total of 181 genes being selected (Figure 2C). Under these stringent conditions, 151 genes containing at least one pG4 located near a PAC were identified, thus confirming the prevalence of pG4 in 83% of PACs under RHPS4 treatment conditions. In order to be able to focus on promising candidates, the selection criteria were further defined by extracting APA events with a PDUI of greater than 12%. Furthermore, cases involving introns, and those with multiple exons containing PACs, were also excluded. As a result, the analysis was narrowed down to 15 genes, which encompassed a total of 27 pG4 structures (Figure S4).

### pG4 near the proximal PAC of NEO1 3′-UTR can form a G4

In the APA dataset, configurations with numerous PACs and numerous identified pG4s can present challenges when selecting a candidate for the proof of concept, as was mentioned earlier. Therefore, it was endeavored to pinpoint an optimal scenario for the subsequent investigations. This process involved identifying a favorable spacing between the two PACs so as to facilitate the straightforward differentiation between the long and short mRNA forms, while also ensuring the presence of no more than two pG4s. The focus was then placed on the potential G4s (pG4) located near the PACs of NEO1. The NEO1 protein is a single-pass transmembrane receptor belonging to the immunoglobulin (Ig) superfamily,^28^ and it plays essential roles in various cellular processes such as cell motility and adhesion (e.g., axon guidance and vascular development),^29^^,^^30^ as well as in survival and differentiation.^31^ In addition to being in a situation that is easy to study and a cancer-related protein, membrane proteins like NEO1 are highly cell-type specific, with differences in 3′-UTR length playing a significant role in their regulation. They are typically regulated by interactions between their 3′-UTRs and RNA-binding proteins (RBPs), which serve to recruit effector proteins to the translation site and could potentially be disrupted by APA-shortened 3′-UTRs.^32^^,^^33^ The modest change in NEO1 expression (0.5983) following ligand treatment was a promising observation. While the effects of RHPS4 on DNA are well-documented, the potential impact of G4 ligands on APA and their influence on transcription were not immediately apparent, which guided our strategic decision to use NEO1 as a proof of concept. Visual representation of the reads obtained for the 3′-UTR of NEO1 revealed the presence of four PolyA cluster (PAC) sites, as determined through DPAC analysis. More precisely, PAC1 and PAC2 that are located near the stop codon (i.e., in the proximal region), and PAC3 and PAC4 that are located in a distal area (PAC are highlighted in black in Figure 3A). This observation suggests the production of two distinct mature RNAs with varying 3′-UTR lengths. Unexpectedly, the treatment with RHPS4 prompted a notable preference for the selection of the distal polyA site, with a PDUI of 55% (as compared to that of 37% of untreated cells), representing an 18% increase of the longer RNA form (Figure 3A). Several *cis*-elements involved in the APA mechanism were also identified: the consensus UGUA motif that is known to be recognized by the CFI complex (highlighted in blue in Figure 3B),^34^ and four specific six-nucleotide PAS sequences (highlighted in gray in Figure 3B) that are typically located 10–40 nts upstream of the polyA cleavage site. Among the 18 PASs curated in the human genome, the distribution of each PAS identified in NEO1 has been determined through motif analysis conducted across four human databases.^35^ Additionally, a U/GU-rich downstream element (DSE) was found to be located approximately 40 nts downstream of the cluster sites (highlighted in orange in Figure 3B). Furthermore, as previously mentioned, the G4RNA screener identified two potential rG4s (represented in red in Figure 3B) located near these PACs based solely on the nucleotide sequence of the RNA. The first, named “proximal rG4,” lies 32 nts downstream of PAC2; while the second, termed “distal rG4,” lies 59 nts upstream of PAC3 (Figure 3B). The proximal rG4 shows a significantly elevated probability of forming a G-quadruplex, with scores of 2.2 for G4 Hunter, 0.99 for G4NN, and 77 for cG/cC. Regarding the distal rG4, it also demonstrates relatively high scores (1.2 for G4 Hunter, 0.8 for G4NN, and 4.3 for cG/cC), suggesting a propensity for rG4 formation, albeit to a lesser extent (Figure 3B).

Using the sequences of the two RNAs corresponding to the proximal and distal rG4s that had been transcribed *in vitro*, fluorescence assays were conducted in order to confirm their actual folding into rG4 structures. The nucleotides located 15–20 nt both upstream and downstream of the G4s were included so as to maintain the natural context of these structures, as neighboring sequences are known to potentially impact the folding of a pG4.^36^ Additionally, the light-up probe N-methyl-mesoporphyrin IX (NMM), which is known to emit fluorescence upon binding to a native rG4 structure, was used.^37^ As anticipated, when binding to the proximal rG4, NMM exhibits fluorescence intensities 40 times greater than that of NMM alone (F/F0), a level which is comparable to that of a characterized rG4.^17^^,^^38^ This finding remains consistent in the presence of both K^+^ (rG4 favorable folding conditions) and Li^+^ (rG4 unfavorable folding conditions), indicating the remarkably high prevalence of this rG4 structure. However, the distal rG4 does not exhibit the ability to enhance fluorescence intensity, irrespective of the salt conditions present in the solution (Figure 3C). This result was not altogether unexpected, considering the positive score achieved by the G4RNA screener for the distal rG4.

### RHPS4 stabilizes both the NEO1 3′-UTR’s distal and proximal rG4 structures

**Figure 4:**
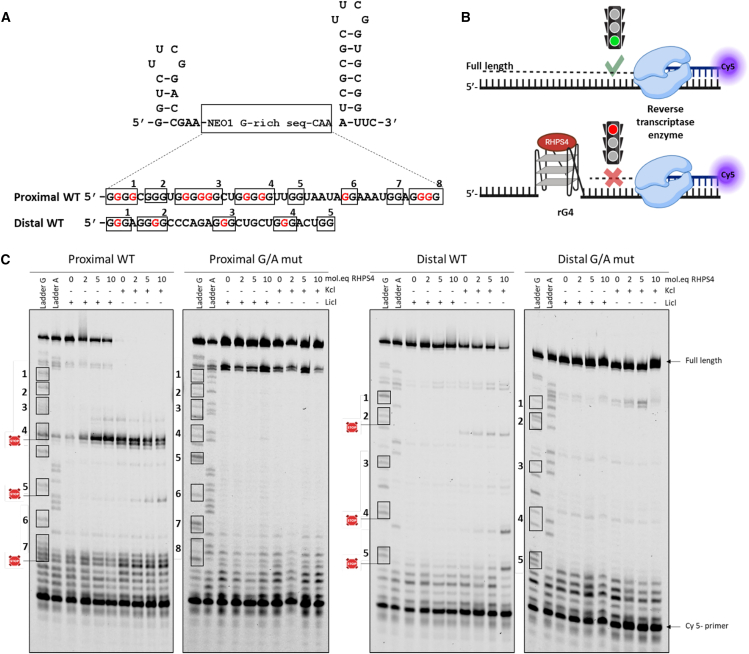
Assessing both the stabilization and the selectivity of RHPS4 on the proximal and distal rG4s of the NEO1 3′-UTR using reverse transcription stop assays (RTS) (A) The sequences of both the proximal and the distal G4 RNAs in their native (wild type) form are capable of folding the rG4 structure, while those with the point mutations from G to A, which are highlighted in red, are unable to fold the rG4 (G/Amut). These sequences were inserted between the two hairpins. (B) Schematic diagram of the RTS reaction performed in the presence of RNA either with or without rG4 stabilization by RHPS4. (C) RTS reactions were analyzed on 10% denaturing polyacrylamide gels. Each RNA was tested in the presence of increasing concentrations of the RHPS4 ligand (0, 2, 5, or 10 molar equivalents relative to the RNA concentration) under conditions that were either favorable (K+) or unfavorable (Li+) for rG4 folding. Lanes labeled with “A” indicate the presence of ddTTP during the reaction, while those labeled with “G” were performed in the presence of ddCTP. All assays were performed in triplicate.

The NEO1 mRNA harbors two G-rich sequences capable of forming natural rG4 structures, as identified by G4RNA screener. The proximal rG4 has been confirmed to form an rG4 *in vitro* without the need for a ligand, while confirmation of the distal rG4 structure may necessitate the use of RHPS4. Considering the significant change in APA observed after RHPS4 treatment, both sequences may function as APA switches. Therefore, it was attempted to determine if RHPS4 could enhance the formation of both the distal and the proximal rG4 structures. In order to achieve this, reverse transcriptase stop assays (RTS-assays) were conducted on both wild-type (WT) sequences and G/A-mutants designed to prevent rG4 formation by replacing certain guanines (G) with adenines (A). RTS-assay was employed to investigate the control of rG4 formation with a G4-ligand during reverse transcription.^16^^,^^17^ Essentially, the reverse transcriptase enzyme either halts, or slows down, when it encounters a stable structure such as an rG4. Consequently, it becomes feasible to detect this interruption using a Cy5-labeled antisense primer by separating the resulting single-stranded DNA on a denaturing gel (Figure 4A). All RNAs (WT-proximal, WT-distal, GA-proximal, and GA-distal) were embedded between a 5′ hairpin, which serves as internal RTS control, and a 3′ hairpin for primer binding (Figure 4B). RTS-assays were carried out in the presence of increasing concentrations of RHPS4 (0–10 mol.equivalent/RNAs) under either Li^+^ or K^+^ conditions (150 mM). For WT-proximal rG4, the band pattern reveals stops from the first nucleotides corresponding to the last stretch of Gs (see box #8 in Figure 4C), as well as a second stop at box #4 under all conditions (with or without RHPS4, in the presence of both Li^+^ and K^+^), thus validating the prevalence of the proximal rG4 as demonstrated by the NMM assay. Conversely, GA-proximal showed no premature stops, even in the presence of 10 mol.equiv/RNAs RHPS4 under the K^+^ condition, confirming the total absence of G4-forming structures in the GA proximal mutant.

In the presence of Li+, there is a notable increase in premature stops following the addition of RHPS4 with WT-proximal. However, the polymerase can still complete polymerization until the end, extending up to the 5′ hairpin, indicating incomplete formation of rG4 at this site (under unfavorable salt conditions). Nevertheless, under the K^+^ condition, the stop profile remains similar, with only the addition of a third stop at box #6 (Figure 4C). The presence of RHPS4 does not seem to affect the intensity of the bands, and the reverse transcriptase fails to complete polymerization, highlighting the significant stability of this structure.

As mentioned elsewhere, the distal rG4 presents an interesting scenario. Despite the scores obtained from the *in silico* analysis of its sequence indicating the presence of an rG4 structure, the results in solution with NMM suggest the opposite, even in the presence of K^+^. Therefore, the assays conducted on the WT sequence under Li^+^ conditions validate the fluorescence observations, indicating no formation of rG4 structures. However, in the presence of K^+^, a faint band is observed at the level of box #2, and the addition of RHPS4 intensifies this band while also causing premature stops to appear at the levels of boxes #4 and #5 with 10 eq RHPS4, indicating the formation of a weak distal rG4 under these specific conditions (K^+^ + RHPS4).

### Mutations that impair distal G4 folding can alter the APA of NEO1

**Figure 5:**
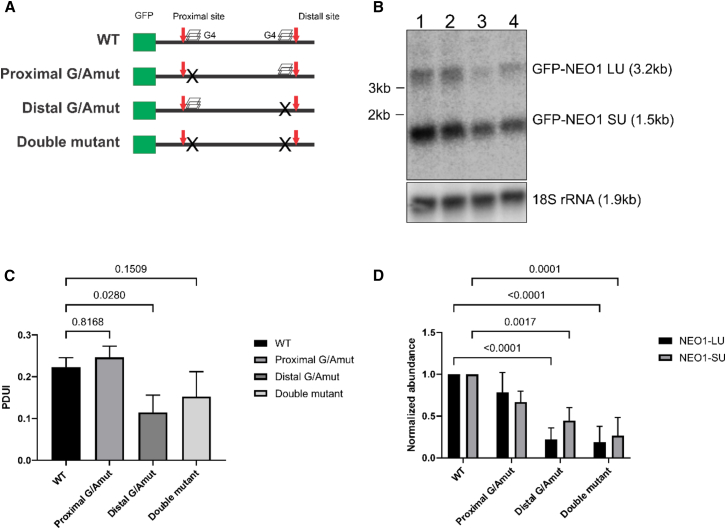
Mutations that prevent the formation of the distal rG4 decrease the use of the distal polyadenylation site (A) Graphical representation of the RNAs transcribed after transfection of the plasmids constructs containing the GFP-NEO1 3′-UTR fusions into HEK-293T cells. In all 4 constructs, the 3′-UTR of NEO1 was fused to the GFP sequence. In the diagram, the positions of both the proximal and the distal polyA sites are indicated by red arrows, while the rG4 sequences are represented by stacked squares. When the latter are mutated, they are represented by Xs. (B) Typical northern blot permitting the detection of both the long (GFP-NEO1 LU) and the short (GFP-NEO1 SU) isoforms using a GFP probe 24 h after transfection. The loading control used was the 18S RNA which can be seen at the bottom. Lane 1, WT; lane 2, proximal G/Amut; lane 3, distal G/Amut; and lane 4, double mutant. (C) The intensities of the GFP-NEO1 SU and LU transcripts were determined from the northern blotting experiments using ImageJ. Then, the PDUI (LU/[LU + SU]) were calculated. The experiments were repeated three times with each of the constructions, and the error bars represent the standard deviations. The statistical analysis performed was a one-way ANOVA with Dunnett’s multiple comparison of each tested construction’s performance versus that of the WT. The adjusted *p* values are shown for each comparison. (D) The intensities of the GFP-NEO1 SU and the LU transcripts were determined from the northern blot hybridizations as in (C), except that the intensities were normalized against those of the 18S RNA (for the loading control) and of the WT isoforms (for abundance). The experiments were repeated three times with each of the constructions being transfected once. The statistical analysis performed was a two-way ANOVA with Sidak’s multiple comparison of each tested constructions performance versus that of their respective WT. The adjusted *p* values are shown for each comparison. The error bars represent the standard deviations.

Following confirmation of the rG4s′ formations, the individual role of each rG4 in APA in cells was explored. Briefly, the full-length 3ʹ-UTR, as well as the GA mutant versions, of the NEO1 gene were cloned downstream of the GFP open reading frame (ORF) in the pEGFP-C1 vector. This procedure included the removal of both the SV40 polyA signal and the synthetic PAS that were already present in the vector. In total, four constructs were created through mutagenesis: one containing the WT NEO1 sequence with both the proximal and the distal rG4 structures, another that was unable to fold the proximal rG4 (GA proximal mutant), third that was unable to fold the distal rG4 (GA distal mutant), and fourth with mutations preventing the formation of any rG4 structures (double mutant, Figure 5A). The transfection of the plasmids into HEK293T cells and the subsequent isolation of total RNA permitted the evaluation of the abundances of both the long (GFP-NOE1-LU at 3.2 kb) and short 3′-UTR isoforms (GFP-NEO1-SU at 1.5 kb) at 24-h post-transfection by northern blot analysis (Figure 5B). After averaging data from three biological replicates, the PDUI for each construct was calculated using the formula (LU/[LU + SU]) (Figures 5C and 5D). In the raw results, the PDUI for the wild-type (WT) 3′-UTR sequence was found to be 0.22 that of LU, which falls within the same range as the PDUI obtained through the DPAC analysis of the endogenous NEO1 gene (36% of LU in untreated cells). This suggests that the long isoform is unfavorable in both instances, indicating a consistent and reliable pattern of APA for NEO1. Surprisingly, the mutation of the proximal rG4 does not drastically change the PDUI (0.25), despite appearing to be the one that is both the most formed in NMM and the most stabilized in RTS. Conversely, mutation of the distal rG4 led to a significant reduction in the production of the long form (PDUI = 0.11). The double mutant exhibited an intermediate level at 0.15, similar to that of the distal mutant. While a significant, direct effect on the APA has been observed, specifically on the PAS, the guanines implicated in the rG4 folding found by RTS and NMM, both for the distal and the proximal rG4, do not correspond to the conventional recognition sites (PAC, PAS, and DSE). Overall, the distal G-quadruplex formation appears to have a direct impact on the polyA site that the RNA processing machinery selects.

## Discussion

For the past decade, the formation of rG4 structures in the 5′-UTRs of genes has been recognized for the crucial role that it plays in translation as it influences ribosome recruitment.^39^^,^^40^ These structures can either act as roadblocks to ribosome progression, or can facilitate the preferential recruitment of the translation machinery.^39^^,^^40^^,^^41^^,^^42^^,^^43^^,^^44^^,^^45^^,^^46^^,^^47^ However, research on rG4 structures located at the ends of transcripts remains limited and anecdotal. While the pG4r density in the 5′-UTR is higher than that in the 3′-UTR across all mRNAs,^26^ our extensive dataset reveals that rG4s located in the 3′-UTR contribute significantly to mRNA maturation. G4s are a prevalent feature of APA within the RNA landscape, as was demonstrated by their identification using a classic pharmacological probe (RHPS4) targeted against the well-characterized rG4 motif. In a general manner, G4-ligands (such as RHPS4) have the potential to stabilize multiple types of G4s and to exert a multifaceted regulatory effect on various G4s, complicating the analysis of their regulatory role in G4-APA. The choice to prioritize the use of RHPS4, rather than other ligands known for their greater thermal stability (such as PDS or phenDC3), was based on its relatively lower “structure-inducing” effects. This trade-off enables an initial selection of events related to the existing G4 structures in the cell, specifically in relation to the ligand itself.

However, through DPAC-seq analysis a correlation between the formation of 3′-UTR rG4s around APA sites and significant changes observed when cells are exposed to RHPS4 was clearly demonstrated. The NEO-1 transcript was identified as an ideal candidate to demonstrate these significant events. More precisely, the formation of 2 rG4s (named the proximal and distal rG4s) located around 2 PACs (located 32 and 59 nt from the PAS, respectively) was discovered using an efficient bioinformatic pipeline. Upon stabilizing the G4s of NEO1 with RHPS4 in cells, an increase in the PDUI (from 0.36 to 0.55) and an elevation in the abundance of the NEO1 transcript were observed (refer to the data analyzed with DPAC and DSeq2, respectively). Additionally, functional assays conducted with northern blot fusion GFP-3′-UTR NEO1 experiments highlighted the significance of the distal rG4. Specifically, when the distal rG4 was mutated a decrease in the PDUI from 0.22 to 0.11 and a reduction in the NEO1 expression level were observed. This decrease did not seem to be due to a loss of stability of the long isoform, as the mRNA stability assay conducted using transcription inhibition by actinomycin D demonstrated (Figure S5). In all scenarios, the anticipated trend persisted: the short isoform (SU) remains more stable as compared to the long one (LU). Nevertheless, in the context of the distal G/A-mut, this stability difference appears to mirror what was observed with the wild-type. To further understand the mechanisms underlying these APA shifts, transcriptomic data were analyzed for the expression levels of canonical APA core regulators required for reconstitution of 3′ end processing APA, including components of the cleavage and specificity factor (CPSF), cleavage simulator factor (CstF), and cleavage factor intermediate (CFIm) complexes.^48^^,^^49^ Specifically, CPSF1-4, CPSF6, CPSF7 (CstF), WDR33, and FIP1L1 (associated with both CPSF and CFIm), NUDT21 and PABN1 were examined. Among these, upregulation of FIP1L1 was identified. FIP1L1 is known to play a dual role in the 3′ processing: as a core component of the CPSF complex, it enhances polyA polymerase activity and polyadenylation efficiency; functionally, it also associate with CFIm subunits to influence polyA site selection.^50^ FIP1L1 overexpression has been linked to preferential usage of distal PAS, often resulting in longer 3′ UTRs.^50^^,^^51^ However, the APA pattern across our dataset revealed 36 events of 3′ UTR shortening, 86 of lengthening, and 59 mixed events, indicating a heterogeneous response. These findings suggest that while FIP1L1 may contribute to a subset of APA lengthening events, the global APA profile is shaped by a more complex interplay of regulatory elements and cellular contexts. This reflects the broader capacity of G4-ligands like RHPS4 to modulate diverse transcriptomic landscapes beyond individual APA regulators.

RHPS4 can also alter DNA G4 structures, and might be linked to the observed changes in APA, prompting consideration of the potential impact of G4 formation within DNA on upstream transcription processes.^16^^,^^52^^,^^53^ Biophysical studies (including CD, NMM, and RT-stop assays) generally indicate minimal to negligible formation of distal DNA G4 structures, as well as lower levels of proximal rG4 structures, as compared to G4 RNA in NMM (see Figures S6 and S7). The results indicate that the addition of RHPS4 has a minor effect on both proximal and distal DNA G4 structures, as demonstrated by the CD spectra and the RTS-assay. Nevertheless, it is worth mentioning that while the rG4 proximal mutation did not change the 3′-UTR’s length (i.e., it is likely unresponsive to RHPS4 addition), the mutation of the distal rG4 presents a noticeable effect on the 3′-UTR’s length and favored the short isoform. Although key elements of PolyA (PAC and PAS) are located close to the mutated sequences, the rG4s are not part of the identified PAS or PACS. This clearly highlights the effect of the rG4 structure in the 3′-UTR region, especially that of the distal rG4, on APA. Recent studies have highlighted the significance of the distal site over the proximal one on 3′-UTR length, indicating a sequential regulation of APA. This involves the initial co-transcriptional selection of the distal PAS followed by the post-transcriptional utilization of the proximal PAS.^54^ Here, the data indicate that an rG4 located near a proximal PAC enhances the usage of the corresponding proximal PASs (pPAS). In the cases of other G4s described as impacting the 3′-UTRs of LRP5 and FXR1, the rG4s are situated downstream of the pPAS and upstream of the distal PAS.^9^ Overall, this spatial arrangement appears to preclude the use of the distally located site, thus causing a decrease of the longer isoforms, as it is also observed for NEO1.

A recent study demonstrated how the formation of secondary structures, such as rG4 and hairpins in the 5′-UTR, can be utilized to achieve a 12-fold increase in translation.^39^ Beyond its role as an mRNA modulator *in cellulo*, this alternative RNA structure could enable more precise control of polyA site regulation through the formation of synthetic G-quadruplexes in the 3′-UTR. This could in turn permit the production of transcripts with higher stabilities, while simultaneously affecting preferential protein isoform production. Thus, this strategy may shift our view of secondary structures from adverse entities to powerful modulators of the proteome, impacting all areas where these molecules have therapeutic and biotechnological potential.

### Limitations of the study

One inherent limitation of PolyA Click-seq analysis is the variability in statistical thresholding practices across studies, which can significantly influence the interpretation of differential expression results. Earlier implementations of Click-seq often used relatively permissive thresholds, such as an adjusted *p* value of <0.10, whereas more recent studies—including Elrod et al. (2020)—have adopted more stringent criteria, such as Bonferroni-adjusted *p* values of <0.05. This evolving methodological landscape reflects the ongoing challenge of balancing sensitivity and specificity in transcriptome-wide analyses. While our chosen threshold aligns with established practices and was defined with expert input, we acknowledge that the selection of statistical cutoffs remains a critical parameter that can influence downstream biological interpretations.

Additionally, the PolyA Click-seq approach presents certain chemical limitations, particularly regarding the use of G4-binding ligands. These compounds, even when chosen to minimize structural induction, may still stabilize or promote G-quadruplex (G4) formation, potentially biasing the observed distribution of G4-containing transcripts away from their native abundance. To mitigate this, we complemented our transcriptomic data with stringent *in silico* predictions using high-confidence G4-scoring algorithms, and validated selected G4 formations *in vitro* using multiple orthogonal techniques. Despite these efforts, this remains an important consideration in the interpretation of our results.

## Resource availability

### Lead contact

Requests for further information and resources should be directed to and will be fulfilled by the lead contact, Pauline Lejault (pauline.lejault@usherbrooke.ca).

### Materials availability

This study did not generate new unique reagents. All unique/stable reagents generated in this study are available from the lead contact without restriction. All unique/stable reagents generated in this study are available from the lead contact with a completed materials transfer agreement.

### Data and code availability

• RNA-seq data (FASTQ files) have been deposited at NIH database: PRJNA1218045.
• All original code has been deposited on GitHub: https://github.com/Ilhyon/APA-G4.git.
• Any additional information required to reanalyze the data reported in this paper is available from the lead contact upon request.

## Acknowledgments

We would like to thank Dr. Anais Vannutelli for her invaluable assistance and insightful discussions during the bioinformatic analyses of the PolyA Click-seq and the G4 identification. We are also grateful to Dr. Andrew Routh, Chief Scientific Officer at ClickSeq Technologies, for his valuable advice during the revision process, his kindness, and the time he dedicated to supporting our work. We also acknowledge our funding sources: this project was supported by grants from the 10.13039/501100000038Natural Sciences and Engineering Research Council of Canada (NSERC; RGPIN-2023-04178) to J.P.P. P.L received a postdoctoral fellowship from the Center de Recherche du Center Hospitalier de l’Université de Sherbrooke (CRCHUS). M.A.T. received student fellowships from the Fonds de Recherche Québec Nature et Technologie (10.13039/501100003151FRQNT) and the Canadian Institutes of Health Research (10.13039/501100000024CIHR). J.P.P. holds the Research Chair of the Université de Sherbrooke in RNA Structure and Genomics. The funders had no role in study design, data collection and analysis, the decision to publish, or in the preparation of the manuscript.

## Author contributions

F.B., P.L., and J.P.P. conceptualized this study; F.B., P.L., and M.A.T. performed the experiments; F.B., P.L., and M.A.T. analyzed the data; and P.L., F.B., and J.P.P. wrote and revised the manuscript.

## Declaration of interests

The authors declare no competing interests.

## STAR★Methods

### Key resources table

**Table:** 

REAGENT or RESOURCE	SOURCE	IDENTIFIER
**Bacterial and virus strains**		

E.coli One Shot™ Stbl3™	Thermo fisher	C737303

**Chemicals, peptides, and recombinant proteins**		

RHPS4 3,11-Difluoro-6,8,13-trimethylquino[4,3,2-*kl*]acridinium methylsulfate	Bio-techne	cat#5311
pyrophosphatase	Roche Diagnostics	N/A
DNase RQ1		
T7 RNA polymerase	Thermo fisher	EP011SKB008
N-Methyl-Mesoporphyrin IX (NMM)	Frontier Scientific Inc., Logan, Utah	NMM580
MMuLV-reverse transcriptase	New England Biolabs	M0253S
Q5 DNA polymerase	New England Biolabs	M0491S
Restriction enzymes *Xho* I and *BamH* I	New England Biolabs	Lot #10161948.

**Critical commercial assays**		

Transcription of RNA radiolabelled (MaxiScript T7 kit)	Invitrogen (supplier fisher)	17984681
Plasmid preparation (DNA purification)	PCR purification kit (Bio Basic)	BS363
Plasmid preparation ((DNA extraction)	DNaesy Blood and Tissue kit (Qiagen)	ID. 69504

**Deposited data**		

RNA sequencing data (FASTQ files) are stored in the NIH database	This paper	PRJNA1218045
All of code is available on GitHub	This paper	https://github.com/Ilhyon/APA-G4.git

**Experimental models: cell lines**		

HEK293T		

**Oligonucleotides**		

Oligonucleotides for CD, NMM and RT-stop assay (see the STAR Methods section and table of oligonucleotides in Supp. figures Table S1)	Integrated DNA technology	N/A
Oligonucleotides for cloning, northern blot (see the STAR Methods section and table of oligonucleotides in Supp. figures Table S1)	Integrated DNA technology and this paper	N/A

**Software and algorithms**		

Geontology (GO)		
G4RNA Screener (G4Hunter (G4H), cG/cC, G4NN)	this paper	http://scottgroup.med.usherbrooke.ca/G4RNA_screener/
All of code is available on GitHub	this paper	https://github.com/Ilhyon/APA-G4.git

### Experimental model and study participant details

Human embryonic kidney 293T (HEK293T) cells (Homo sapiens; ATCC® CRL-3216™) were used in this study. HEK293T cells are an immortalized human cell line derived from embryonic kidney tissue of a male donor and genetically modified to stably express the SV40 large T antigen. As such, they represent a single genetic background, with limited applicability across sexes, ancestries, races, or ethnicities; Cells were maintained in Dulbecco’s Modified Eagle Medium (DMEM) supplemented with 10% fetal bovine serum (FBS) and cultured under standard conditions (37 °C, 5% CO_2_) according to the supplier’s recommendations. Regular mycoplasma testing was performed to ensure culture quality.

### Method details

#### Genetic material

All Oligonucleotides used in this study were purchased from Integrated DNA technology (IDT) and were stored at −20°C as 100 μM stock solutions in deionized water. The actual concentration of these stock solutions was determined by spectrophotometry at 260 nm (see the table of oligonucleotides in Supp. figures Table S1). RHPS4 was purchased from Bio-techne and was dissolved in water at a concentration of 10 mM prior to storage at 4°C. When required, these stock solutions were then diluted with deionized water to the appropriate concentration for the experiment in question.

#### Dose–response of RHPS4

Human embryonic kidney HEK-293T cells cultured in Dulbecco's modified Eagle's medium (Wisent) supplemented with 10% fetal bovine serum (Wisent) were seeded at 5 000 cells/well in a 96-well flat bottom plate. The next day, the cells were treated with a series of RHPS4 (#5311, Bio-Techne) solutions of different concentrations made by serial dilution of the stock solution. After incubating for 72 h, cell proliferation assays were performed using an MTT assay kit (Abcam) according to manufacturer’s recommendations. Three biological replicates were carried out in order to generate the dose-response curve from which the inhibitory concentrations (IC_15_, IC_25_, IC_50_) were calculated (Figure S1).

#### PolyA click-seq

HEK-293T cells were seeded at 5x10^5^ cells in a 10 cm petri dish (DMEM medium, see above). The next morning, RHPS4 was added to a final concentration of 1.5 μM and the cells incubated for 72 h at 37 °C in a humidified, 5% CO_2_ atmosphere-controlled incubator. In parallel, a non treated petri dish was also incubated for an equivalent time period. Total RNA was then isolated using Qiazol according to the manufacturer’s protocol. Following dissolution in high pure water, the total RNA was further purified using RNAspin Mini isolation kit (Cytiva) that includes on-column DNAse I digestion, all according to manufacturer’s protocol. Total RNA was eluted, its concentration determined by spectrometry at 260 nm using a NanoDrop spectrophotometer (Thermo Fisher Scientific, Mississauga, ON) and its quality assessed by agarose gel electrophoresis. Three biological replicates were carried out before sending these samples to the company Click-seq Technologies LLC (Davis, CA, USA) in order to analyze the changes in polyadenylation by PolyA Click-seq. Library preparation and sequencing were performed as described previously.^19^ The data analysis pipeline was performed using the DPAC software for the polyA changes and DESeq2 for the gene expression variations, as described previously.^55^ Single-end run (150 bases) was performed on an Illumina NextSeq 550 sequencer. The reads were then mapped on the Homo Sapiens genome assembly (hg19). The results were returned in an Excel file. Using the program DPAC, the poly(A)-Sites (PASs) were defined as the exact nucleotide of the 3’-UTRs with at least 5 mapped reads and a poly tail with 25 Adenines (minimum). All provided samples were used to generate a project specific database of PolyA-Clusters (PACs), which was subsequently used for both the differential gene and the alternative polyadenylation analyses. PACs were defined as groupings of polyA-sites that are located within 10 nucleotides of one another. Gene counts for each sample were generated using DPAC. Using the newly generated annotated PAC database, the 3’ end of all of the mapped reads overlapping with PACs identified contributed to that gene’s count (within a given gene). The count table was then used as the input into DESeq2 for the differential gene expression analysis. Differential gene expression was defined as a >1.5-fold change with a p-adjust <0.1. If there are more than one PolyA-Cluster in a given 3’-UTR, and one or more of them are 'significantly' regulated following the addition of RHPS4, then it can be said that there is either “lengthening” or “shortening” APA (according to DESeq2 and DPAC) if there is a change in any 3' end’s alternative isoform of greater than 10% (p-adjust <0.1). Moreover, for each significantly regulated event, a differential percentage of distal polyA site usage (ΔPDU) was also generated. The latter considers the length of the 3’-UTRs in each isoform, and weights the contribution of a PAC based upon its genetic distance from the most proximal PAC (no statistical test was involved here). The raw RNA sequencing data (FASTQ files) are stored in the NIH database, under reference number PRJNA1218045. The data are categorized into two groups: non-treated samples (three replicates) and treated samples (three replicates of HEK293T cells treated with RHPS4).

#### Bioinformatics analyses for the presence of potential G4s located near PACs

For each significant event detected, depending on the location of the PACs on the mRNA, there are different types of polyA events that can occur (see Figure 1). In order to simplify the analysis, the search for events that could involve G-quadruplexes was restricted to that called “APA-exon” where the PACs involved are located exclusively in the last exon, that is to say in the 3’-UTR. Thus, for each PAC involved in a significant polyA change following RHPS4 treatment (called by DPAC “lengthening”, “shortening”, or “both”), their chromosomal location was used in order to search for the presence of rG4 located up to 100 nt both upstream and downstream of the PAC. In order to accomplish this, G4 RNA screener, which contains three scoring systems that are used to describe the likelihood of G4 observation (the cG/cC, G4Hunter (G4H) and G4NN scores), was used. For this study, sequences that were considered as being positive implied the obtention of at least one score out of the three that was higher than the following limits: G4H > 0.9, G4NN > 0.5 and cG/cC > 4.5 (the default values of the software) Subsequently, for each PAC positive for the presence of a potential G4 sequence, another level of control was added by deleting all situations for which the ΔPDU was less than 0.12. Finally, some hits were manually cleaned by removing false positive sequences generated by the cG/cC score alone. All of code is available on GitHub: https://github.com/Ilhyon/APA-G4.git.

#### Gene ontology

The Gene Ontology analysis was performed on the list of genes that were either significantly up- or down-regulated by RHPS4 addition. The Gene Ontology term analysis was conducted using the website https://geneontology.org/ in accordance with the GO Ontology database released 2023-05-10, https://doi.org/10.5281/zenodo.7942786.

#### Fluorescence assays

RNA molecules were synthesized by *in vitro* transcription using purified T7 RNA polymerase. Briefly, two oligonucleotides (2 μM each, Life Technologies) were annealed together through their complementary regions, and then purified Pfu DNA polymerase was used in PCR reactions in the presence of 5% DMSO in order to fill in the gaps. The resulting duplex DNA products were then ethanol-precipitated, washed with 70% ethanol, dried and dissolved in ultrapure water. Run-off transcriptions were then performed in a final volume of 100 μL using purified T7 RNA polymerase in the presence pyrophosphatase (0.01 U, Roche Diagnostics) and 5 mM concentrations of each nucleotide triphosphate (ATP, CTP, GTP and TTP) in a buffer containing 80 mM HEPES-KOH (pH 7.5), 24 mM MgCl_2_, 40 mM dithiothreitol (DTT) and 2 mM spermidine. The reactions were incubated for 2 h at 37°C, followed by DNase RQ1 (Promega, Madison, WI) treatment (2 U) for 30 min. at 37°C. The RNA was then purified by phenol:chloroform extraction and recovered by ethanol precipitation. The RNA products were then fractionated by denaturing (8 M urea) 8% polyacrylamide gel electrophoresis. The RNAs in the gels were detected by ultraviolet shadowing, and the bands corresponding to the appropriate sizes were excised from the gels. The transcripts were then eluted overnight at room temperature in a buffer containing 1 mM EDTA, 0.1% sodium dodecyl sulfate and 0.5 M lithium acetate and then were ethanol-precipitated, dried, dissolved in water and their concentrations were then determined by spectrometry at 260 nm using a NanoDrop spectrophotometer (Thermo Fisher Scientific, Mississauga, ON). In order to evaluate G4 formation, *in vitro* synthesized RNAs (200 pmol) were added to a folding buffer containing 20 mM Li-cacodylate pH 7.5, 20 mM MgCl_2_ and 100 mM of either LiCl or KCl and were then heated at 70°C for 5 min before being slowly cooled to room temperature over a period of 1 h. The reactions were then completed to 100 μL with buffer containing 20 mM Li-cacodylate pH 7.5, 20 mM MgCl_2_ and 100 mM of either LiCl or KCl). Next, 2.5 eq/RNA of N-Methyl-Mesoporphyrin IX (NMM) (Frontier Scientific Inc., Logan, Utah) was added, and the reactions incubated in the dark for 5 min at room temperature in a 10 mm quartz cuvette. The fluorescence intensity was then monitored using a Hitachi F-2500 fluorescence spectrophotometer with an excitation wavelength of 399 nm, and the emission spectra were recorded between 500 and 650 nm. The fluorescence at 605 nm was used for quantification. All experiments were performed in duplicate.

#### Reverse transcriptase stops assays (RTS-assays)

RNAs to be tested by RTS were embedded between a 5’ hairpin, that served as an internal RTS control, and a 3’ hairpin that was used for primer binding (Figure 5B).^56^ All reactions were performed both under conditions favorable for G4 folding (KCl) and under ones unfavourable for G4 folding (LiCl). *In vitro* synthesized RNAs were generated as described earlier (see the Florescence assays section) through oligonucleotides annealing and gap filling using a T7 promoter oligonucleotide in a PCR reaction followed by *in vitro* transcription of the double-stranded DNA templates using T7 RNAP. The resulting RNAs were quantified by NanoDrop as previously described, and 4.4pmol were added to 5 pmol of a Cy5 fluorescently labelled DNA primer that was complementary to the 3’ hairpin. The reaction was completed by the addition of 5 μL of binding buffer (100 mM Tris-HCl pH 8.3 and 150 mM of either KCl or LiCl) and 1 μL of 0, 2, 5 or 10 molar-equivalents of RHPS4. The temperature of the mixture was then increased to 75°C for 3 min and then lowered to 37°C and kept there for 5 min in a thermocycler. At the beginning of the 37°C step, 0.5 μL of 10 mM dNTPs, 1 μL of 100 mM DTT, 1 μL of 25 mM MgCl_2_ and 1 μL of water were added. For each of the RNAs tested, 1 mM ddCTP and ddTTP were added to reactions containing LiCl, but not those containing KCl, in order to generate ladders. The 12 μL mixture was heated to 45°C, and 0.5 μL (100 U) of purified MMuLV-reverse transcriptase was then added. The reverse transcription reaction was kept at 45°C for 15 min, at which point 0.5 μL of 2M NaOH were added. The temperature was immediately increased to 90°C for 10 min in order to inactivate the enzyme and degrade the RNA template. Finally, 15 μL of loading dye (98% formamide, 10 mM EDTA) was added and 6 μL of the resulting solution was analysed on a 10% polyacrylamide gel containing 8 M urea. The final images were generated using a GE Typhoon FLA 9000 fluorescence scanner (λex = 635 nm).

#### Plasmids and cloning

Before the cloning of the NEO1 3’-UTR into the pEGFP-C1 plasmid, the synthetic SV40 polyadenylation signal sequence located from positions 1519-1640 of the vector had to be removed in order to prevent any bias that could result from unwanted competition between polyA sites in the NEO1 3’-UTR and the SV40 polyA sequence. Plasmid pEGFP-C1 was used as a template in a PCR reaction with Q5 DNA polymerase (New England Biolabs) and the oligonucleotides 5’-ACGCGTAAATTGTAAGCG-3’ and 5’-AACAACAACAATTGCATTCATTTTATG-3’ in order to generate a DNA fragment lacking region 1519-1640 of the plasmid. The PCR product was then purified using a PCR purification kit (Bio Basic) according to manufacturer’s recommendation. After a KLD reaction (Kinase, ligase and *Dpn* I treatment), the resulting circularized plasmid (called pEGFP-C1 ΔSV40) was transformed into *E. coli* STBL3. Deletion of the synthetic SV40 polyA signal sequence was further confirmed by DNA sequencing. A “STOP” codon was also introduced at the end of the GFP sequence. In this case, plasmid pEGFP-C1 ΔSV40 was used in a Quick-change reaction using a pair of complementary oligonucleotides. The resulting nicked-plasmid (called pEGFP-C1 ΔSV40-Stop) was then transformed into *E. coli* STBL3. The introduction of the STOP codon (TAA) downstream of the GFP open reading frame was confirmed by DNA sequencing. NEO1 3’-UTR was prepared from genomic DNA isolated from cultured HEK-293T cells using the DNaesy Blood and Tissue kit (Qiagen) according to manufacturer’s protocol. Purified genomic DNA (135 ng) was used as a template. The 3’-UTR of NEO1 (2 654 bp) was introduced into linearized pEGFP-C1 ΔSV40-Stop using the restriction enzymes *Xho* I and *BamH* I and the HiFi DNA Assembly Cloning Kit (New England Biolabs) according to manufacturer’s instructions. All of the mutants were created using the same protocol. The presence of the mutations was confirmed by DNA sequencing following the transformation of the ligation products into bacteria (XL1-blue *E. coli*) for amplification and purification.

#### Northern blot hybridization

In order to generate the final 320 nt GFP RNA probe, the DNA template for the *in vitro* transcription was amplified from the pEGFP-C1 construct using the primers 5’-taatacgactcactataGGGTTACTTGTACAGCTCGTCCATG-3’ and 5’-CACAAGCTGGAGTACAACTAC-3’ with Taq DNA polymerase, where the lower-case nucleotides indicate those that correspond to the T7 promoter sequence. After PCR amplification, *Dpn* I (1 μL) was added to the reaction in order to degrade the original pEGFP-C1 template, and the reaction was incubated at 37°C for 1 h. Phenol-chloroform extraction followed by ethanol precipitation was then performed in order to purify the DNA probe. The resulting DNA pellet was dissolved in water and its concentration determined by spectrometry at 260 nm using a Nanodrop spectrophotometer. The 80 nt 18S probe was generated using the antisense oligonucleotide 5’-CCTGGCGGAGCGCTGAGAAGACGGTCGAACTTGACTATCTAGAGGAAGTAAAAGTCGTAACAAGGTTTCCGTAGGTGAACCCtatagtgagtcgtatta-3’ and the sense T7 promoter oligonucleotide as described previously for the GFP probe, except that the PCR product was purified using the PCR purification kit (Bio Basic) according to the manufacturer’s protocol. The probe DNA was eluted with pure water and its concentration determined by spectrometry at 260 nm using a Nanodrop spectrophotometer. The ^32^P-radiolabelled RNA probes were finally synthesized using the MaxiScript T7 kit (Invitrogen) according to the manufacturer’s protocol. Following transcription, the probes were purified using MicroSpin G50 columns (Cytiva) according to the manufacturer’s protocol and were added to pre-hybridization buffer.

HEK-293T cells were seeded at 5x10^5^ cells/well in a 6-well plate. The next day, DNA plasmids (1 μg) containing NEO1 3’-UTR-WT, NEO1 3’-UTR-G/Amut proximal, NEO1 3’-UTR-G/Amut distal and the double mutant fused to GFP were transfected into the cells with 3 μL of 1 mg/mL of polyethylenimine (PEI). Following an incubation of 24 h, total RNA was prepared using 1 mL of Qiazol (Qiagen) per well essentially described in the section “PolyA click-seq”. Again, the concentration was determined by spectrometry at 260 nm using a NanoDrop spectrophotometer (Thermo Fisher Scientific, Mississauga, ON) and the quality was assessed by agarose gel electrophoresis.

In order to perform Northern blot analysis, 7.5 μg of total RNA was dissolved in 30 mM Tricine, 30 mM triethanolamine, 50% formamide, 0.4 M formaldehyde, 0.5 mM EDTA pH 8.0 solution before denaturing the RNA by heating at 70°C for 5 min. The RNAs were separated on denaturing 1% agarose gels containing 30 mM Tricine, 30 mM triethanolamine and 0.4 M formaldehyde at 6V/cm in migration buffer (30 mM Tricine, 30 mM triethanolamine). The RNA was then transferred to Hybond-N+ membrane (Cytiva) 0/N by capillarity in 20X SSC (3 M NaCl and 0.3 M sodium citrate). After UV-crosslinking, the membrane was pre-hybridized in buffer containing 50% formamide, 5X SSC, 1% SDS, 5% Denhardt’s solution and 100 μg/mL salmon sperm DNA for 4 h at 65°C. Internally ^32^P-radiolabelled RNA probe complementary to the GFP sequence was then added and the membrane was incubated O/N at 65°C. Three washes of 15 min each using 1X SSC, 0.1% SDS and one wash of 0.1X SSC, 0.1% SDS were performed at 65°C before exposition of the membrane to a phosphoscreen and detection on a GE Typhoon FLA 9000 scanner. The membrane was reused for probing with the 18S RNA (for loading control) using the same protocol as with the GFP probe. The Image J software was used to measure the band intensities, and GraphPad Prism was used to generate the histograms.

#### Actinomycin assays

Stability tests in the presence of actinomycin D were carried out as described above under “Northern blots”. The difference was that 24 h after transfection of the plasmids, actinomycin D dissolved in DMSO (at a final concentration of 6 μg/mL) was added to each well and the cells were incubated for 0 to 8 h. At each time point, the RNA was isolated by Qiazol (Qiagen) according to the manufacturer’s protocol and was analyzed by Northern blot as described above.

#### Primer extension

DNA with a 3’ hairpin (750 nM) was incubated with a ^32^P-radiolabelled reverse primer (750 nM) in a solution containing 20 mM Li-Cacodylate (pH 7.5) at 95°C for 5 min (oligonucleotides sequences described in Figure S7). The reactions were then cooled down to room temperature at a rate of 1 °C/min. The samples were then diluted in order to obtain final concentrations of 50 mM Tris (pH 7.6), 1 mM dNTPs, 1 mM MgCl_2_, 5 mM DTT, 100 mM KCl/LiCl and 675 nM ligand (thus diluting the RNA to 75 nM). The samples were then incubated at 37°C for 5 min. An in-house prepared Taq polymerase (2.5 U) was then added, and the reaction was further incubated at 37°C for 30 min. The ladders were prepared in a PCR machine. Briefly, a solution containing 55 μM of the DNA containing the 3’ hairpin, 55 μM of the radiolabeled primers, Thermopol buffer (NEB), 0.15 % Triton, 2 U of Vent® (exo) DNA Polymerase (NEB), 54 μM ddGTP, 5.4 μM dGTP, 4.5 μM dATP and 15 μM of both dTTP and dCTP was incubated for 25 cycles of 1 min at 95°C, 1 min at 52°C and 1 min at 72°C. The reactions were stopped by the addition of alkaline formamide loading dye (two volumes of 90 % formamide, 10 mM NaOH). The reactions were fractionated by electrophoresis on denaturing (8M urea) 10% polyacrylamide gels. The gels were dried, exposed to a phosphor screen and visualized on a Typhoon FLA 9000 fluorescence scanner. All assays were performed in duplicate.

#### CD analysis

The DNA samples were dissolved at a concentration of 4 μM in a solution containing 100 mM of either KCl or LiCl, 20 mM of Li-cacodylate (pH 7.5) and either with or without the presence of RHPS4 (10 eq). The samples were then heated at 90°C for 5 minutes and then were slowly cooled down to room temperature. The experiments were performed in a quartz cell with a path length of 1 mm on a Jasco J-810 spectropolarimeter. Data were recorded from 350 to 210 nm at 20°C. All CD spectra were smoothed (4 spectrum accumulations) and analyzed using GraphPad Prism.

### Quantification and statistical analysis

All quantification and statistical analyses were performed using GraphPad Prism and R, as appropriate. For dose–response experiments involving RHPS4 in HEK-293T cells, MTT assays were conducted in triplicate biological replicates, and inhibitory concentrations (IC15, IC25, IC50) were derived from nonlinear regression analysis of normalized cell viability curves (see Figure S1). For high-throughput transcriptomic analyses (PolyA Click-seq), three biological replicates per condition (treated and untreated) were used. Differential gene expression and polyadenylation changes were analyzed using DPAC and DESeq2, and statistical significance was defined as adjusted *p* < 0.1 with a fold change threshold >1.5. Alternative polyadenylation events were called based on ≥10% isoform usage shift (ΔPDU), with manual curation and an additional filter (ΔPDU ≥ 0.12) applied to eliminate borderline or potentially artifactual events. For fluorescence and reverse transcriptase stop assays, technical duplicates were used, and representative data were quantified from independent experiments. Primer extension and CD spectroscopy experiments were repeated at least twice, with quantification performed on smoothed curves and gel band intensities analyzed using ImageJ or GraphPad Prism. For Northern blot and actinomycin D assays, signal intensities were measured using ImageJ and normalized to loading controls (18S rRNA), with results from three biological replicates presented as mean ± standard deviation (SD). The definition of center and dispersion (mean, SD, or SEM) is provided in the figure legends or methods, as appropriate.

### Additional resources

• Requests for further information and resources should be directed to and will be fulfilled by the lead contact, Lejault Pauline (pauline.lejault@usherbrooke.ca).

## Supplemental information

Document S1. Figures S1–S7 and Table S1

Table S2. DPAC NT vs. RHPS4 treated total list

Table S3. DEG list

Table S4. APA splicing list

Table S5. APA list
