# Supplementary material for: G-quadruplexes in the proximity 3′-UTR enhances alternative polyadenylation of Neogenin 1
Source: iScience. 2025 Nov 10;28(12):113952. doi: 10.1016/j.isci.2025.113952 (PMC12686709; doi:10.1016/j.isci.2025.113952)
Supplement: Document S1. Figures S1–S7 and Table S1 [file mmc1.pdf]

iScience, Volume *28*

Supplemental information

G-quadruplexes in the proximity 3′-UTR enhances alternative polyadenylation of Neogenin 1

Pauline Lejault, François Bolduc, Marc-Antoine Turcotte, and Jean Pierre Perreault

Name	Sequence (5' - 3')*
NEO1 G4-proximal (up)	TAATACGACTCACTATAgGCTTTCTGTGCAGTTTCAGTATTGGGGCGGGTGGG GGGCTGGGGGTTGG
NEO1 G4-proximal (down)	ACATTCACAGGACCTCAGCAGCCCCTCCATTTCCTATTACCAACCCCCAGCCCCC CACC
NEO1 G4-distal (up)	TAATACGACTCACTATAgGTCTGTCGCCGCACTGCCTGTGGGAGGGGCCCAGA GGGCTGCTG
NEO1 G4-distal (down)	AGGCCAAACAAGTGTGTACAGACGCCAGTCCCAGCAGCCCTCTGGGCCCCTC
Reverse primer for RTS	**Cy5**/GAACCGCACCGAAGCGCG
NEO1 WT-proximal for RTS	GAACCGCACCGAAGCGCGATTTGCCCCTCCATTTCCTATTACCA ACCCCCAGCCCCCCACCCGCCCCTTGGTCCGAAGACCTATAGTG AGTCGTATTA
NEO1 GA-proximal for RTS	GAACCGCACCGAAGCGCGATTTGCTTCTCCATTTCTTATTACCA ACTCTCAGCTTCTCACTCGTCTCTTGGTCCGAAGACCTATAGTG AGTCGTATTA
NEO1 WT-proximal for RTS	GAACCGCACCGAAGCGCGATTTGCCAGTCCCAGCAGCCCTCTG GGCCCCTCCCTTGGTCCGAAGACCTATAGTGAGTCGTATTA
NEO1 GA-proximal for RTS	GAACCGCACCGAAGCGCGATTTGCCAGTCTCAGCAGCTCTCTG GGCTCCTCTCTTGGTCCGAAGACCTATAGTGAGTCGTATTA

Sense or antisense T7 promoter sequence
3' hairpin sequence
5' hairpin sequence
*Small cap "g" indicates the presence of an added guanosine in the sequence in order to increase the transcription efficiency. This "g" is not part of the natural mRNA sequence.

Supp. Table 1: the oligonucleotide sequences

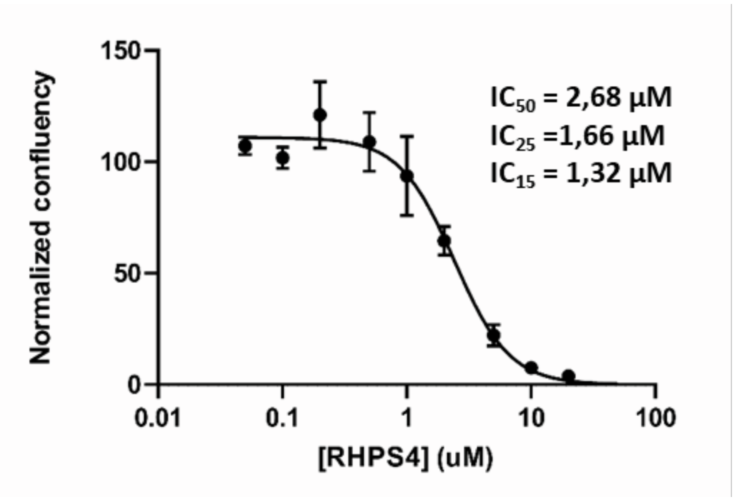

Supp. Fig 1: Dose–response curve of RHPS4 in HEK-293T. The dose response profile for RHPS4 in HEK-293T cells generated from the MTT assays. The experiments were conducted with three biological replicates. The IC_{50}, IC_{25} and IC_{15} are listed in the table below. The error bars represent the standard error of the mean.

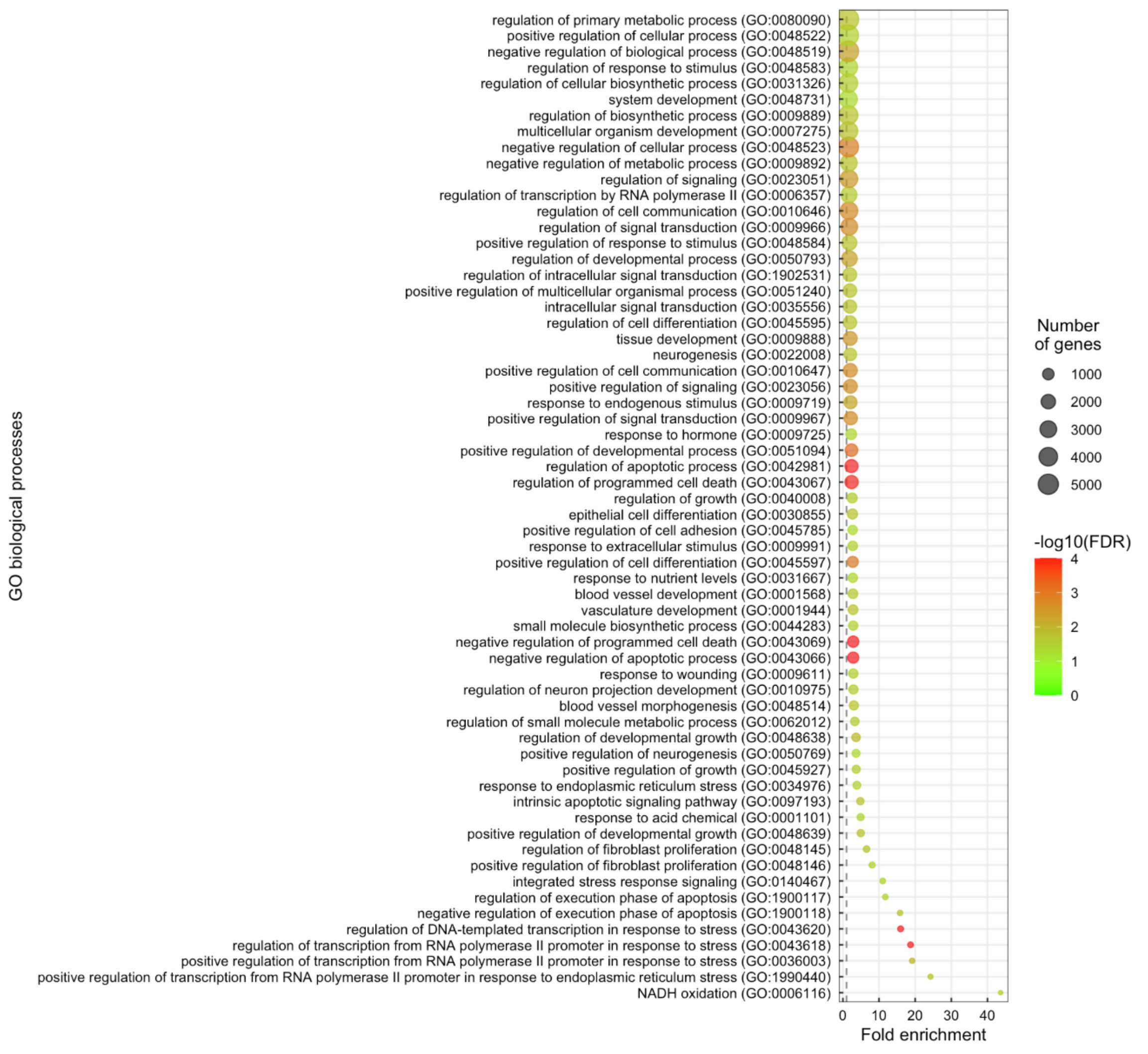

Supp. Fig 2: Gene ontology for the genes that are up-regulated when the cells are treated for 72 h with RHPS4.

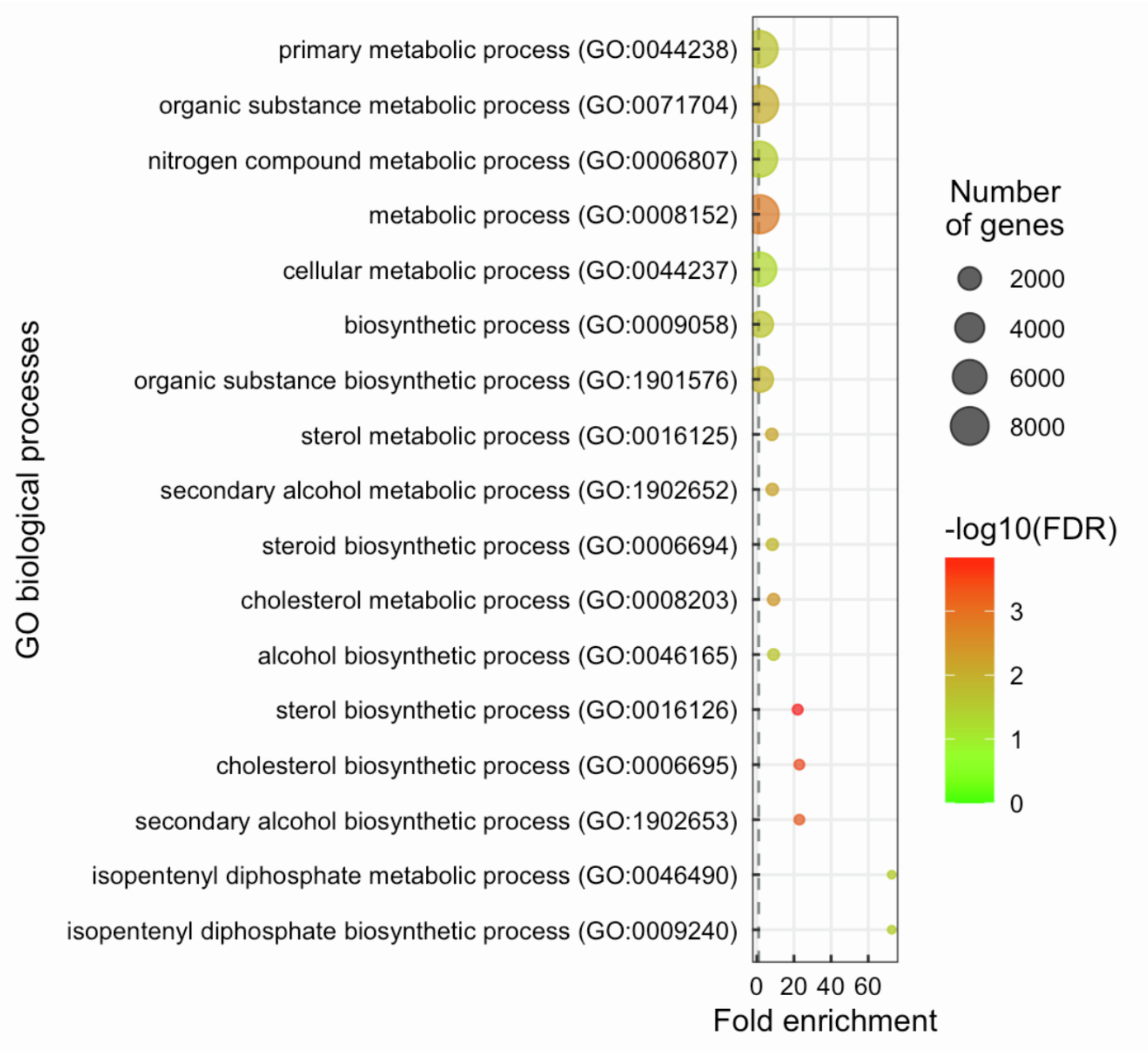

Supp. Fig 3: Gene ontology for the genes that are downregulated when the cells are treated for 72 h with RHPS4.

Gene Name	Gene name Long	#PG4	∆PDU	DPAC Result	Protein function
**ADCY5**	Adenylate Cyclase 5	4	-0,3543	Shortening	Membrane-bound adenylyl cyclase enzymes
**RHOBTB2**	Rho Related BTB Domain Containing 2	1	-0,2126	Shortening	Localized to the Plasma membrane (small Rho GTPase)
**LRP8**	LDL Receptor Related Protein 8	3	-0,1206	Shortening	LDL receptor related protein 8 (Protein predicted to be secreted)
**PECR**	Peroxisomal Trans-2-Enoyl-CoA Reductase	1	0,1216	Lengthening	Participates in chain elongation of fatty acids, Has no 2,4-dienoyl-CoA reductase activity (peroxysomes)
**THAP5**	THAP Domain Containing 5	2	0,1324	Both	DNA-binding, Repressor (nucleoplasm)
**CGRRF1**	Cell Growth Regulator With Ring Finger Domain 1	1	0,1392	Lengthening	Cell cycle, Growth arrest (nucleoplasm and RE)
**TEAD2**	TEA Domain Transcription Factor 2	1	0,1475	Lengthening	Transcription factor (nucleoplasm)
**TLCD5 (TMEM136)**	TLC Domain Containing 5	1	0,1719	Lengthening	Transmembrane protein 136
**RCHY1**	Ring Finger And CHY Zinc Finger Domain Containing 1	1	0,1724	Both	Mediates E3-dependent ubiquitination and proteasomal degradation of target proteins (everywhere)
**SPCS2**	Signal Peptidase Complex Subunit 2	2	0,1767	Lengthening	Component of the microsomal signal peptidase complex which removes signal peptides from nascent proteins (plasma membrane, RE)
**LGR4**	Leucine Rich Repeat Containing G Protein-Coupled Receptor 4	1	0,1798	Lengthening	Receptor for R-spondins that potentiates the canonical Wnt signaling pathway and is involved in the formation of various organs
**GJC1**	Gap Junction Protein Gamma 1	2	0,1803	Lengthening	Component of gap junctions
**NEO1**	Neogenin 1	2	0,1821	Lengthening	Multi-functional cell surface receptor regulating cell adhesion
**HES7**	Hes Family BHLH Transcription Factor 7	2	0,2549	Lengthening	Transcriptional repressor
**ARL4C**	ADP Ribosylation Factor Like GTPase 4C	3	0,3226	Lengthening	Small GTP-binding protein (plasma membrane ,cytoplasm)

Supp. Fig 4: Candidate genes having at least one pG4 located near a PAC. List of the genes for which a significant variation in APA was recorded and which contain at least one pG4 located in the environment of PACs as detected by DPAC. For each gene the table compiles the number of pG4s involved nearby that can influence the APA, the ∆PDUI (the "long 3′UTR expression" divided by the "long 3′UTR expression + short 3′UTR expression"), the result of the DPAC software and the function of the associated protein.

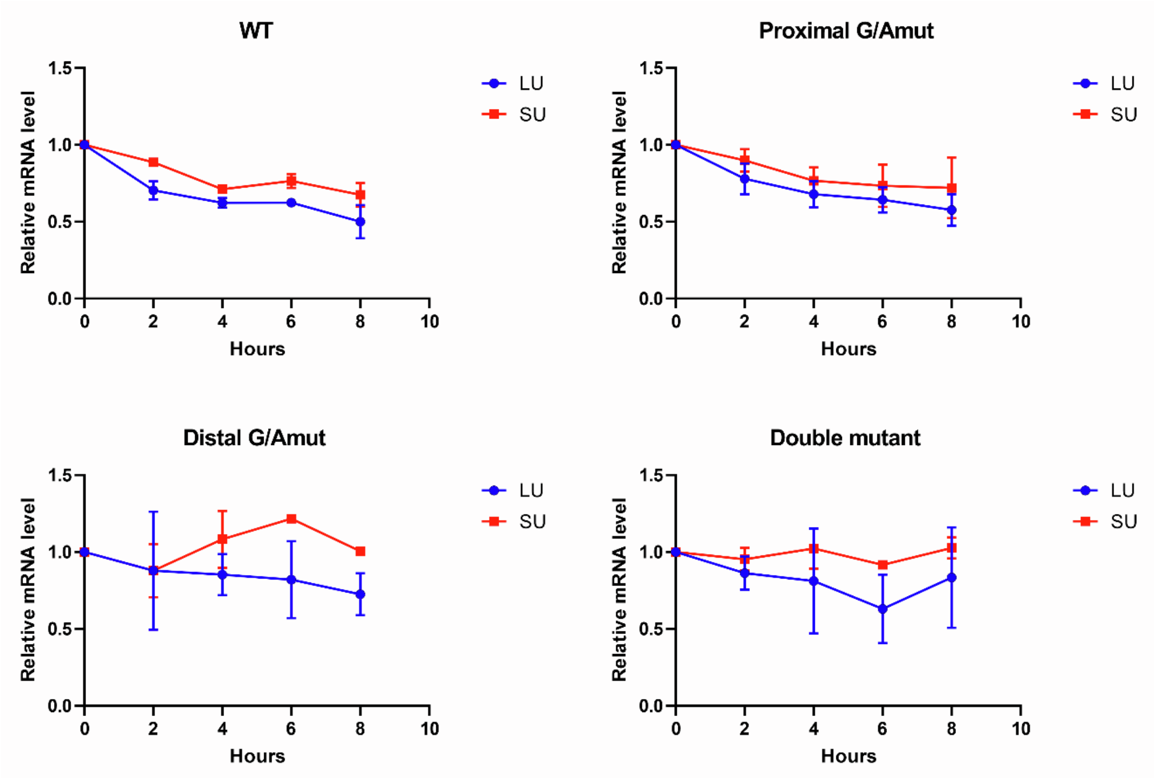

Supp. Fig. 5: Stability assays with actinomycin D. Each plasmid construct containing the GFP-NEO1 3'-UTR fusions and the various mutants (WT, proximal, distal and double mutant) was transfected into HEK-293T cells and, 24 h post-transfection, actinomycin D was added. After 0, 2, 4, 6 and 8 h of incubation, total RNA was isolated. The latter were analysed on a denaturing agarose gel (0.4 M of 37% of formaldehyde) in order to carry out Northern blots with a GFP probe. The intensities of the bands (long (LU) and short 3'-UTR (SU)) were determined using the Image J software and using the 18S RNA as a loading control. These graphs represent the results of two biological replicates.

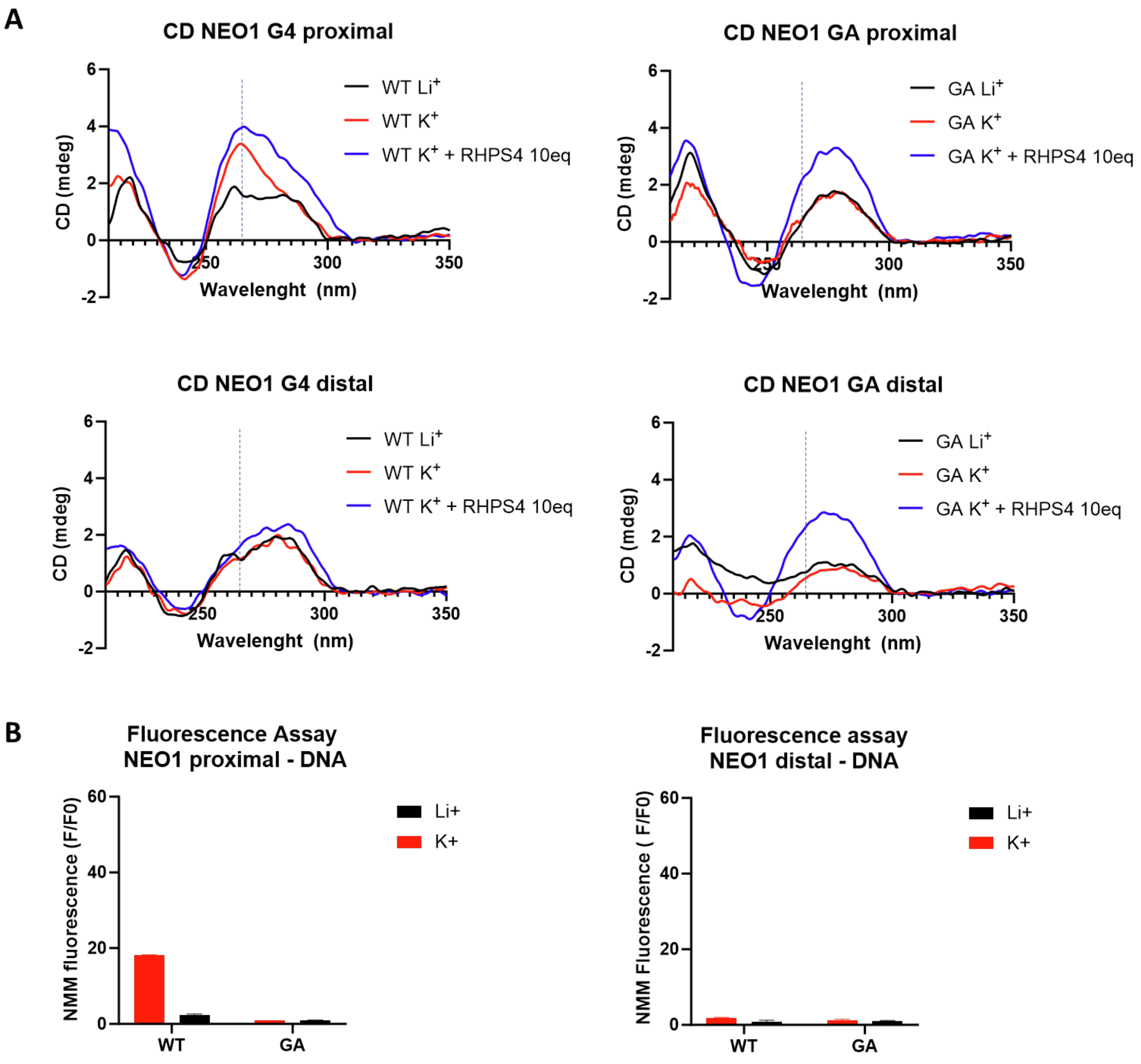

Supp. Fig. 6: *In vitro* characterization of the DNA G4s corresponding to the proximal and distal dG4 identified in NEO1 mRNA using both fluorescent assay and circular dichroism. A) The circular dichroism spectrums were obtained in the presence of 4 µM of DNA and 100 mM of potassium either with or without RHP4 (10 molecular equivalents). B) NMM fluorescence assays were performed in the presence of 2 µM of DNA, 4 µM of NMM and 100 mM of either KCl or LiCl at room temperature. All experiments were performed in duplicate and were analysed by GraphPad Prism.

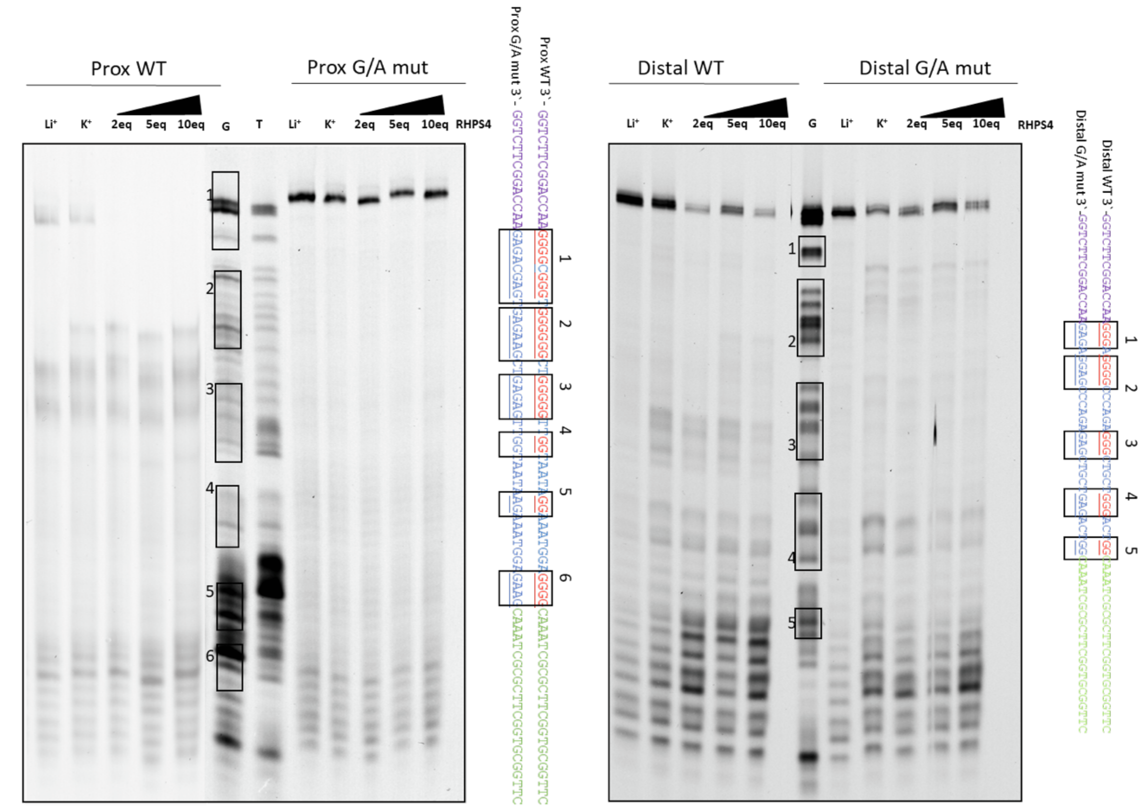

Supp. Fig. 7: Evaluation of the RHPS4-induced stabilization effects on DNA proximal and distal dG4 structures using primer extension assays (PE). The guanine-rich DNAs (WT) or their mutated versions corresponding to identified distal and proximal RNA G4, each with a 3' hairpin (750 nM), were submitted to PE assays using an in-house prepared Taq polymerase (2.5 U) for 30 min at 37°C. The sequences' details are provided in the figure, where each guanine box is highlighted in red and is numbered, the 3' hairpins are highlighted in violet and the 5' hairpins are highlighted in green. The reactions were performed in the presence of increasing amounts of RHPS4 (2 to 10 mol.eq) and used a ^{32}P-labelled primer in buffer containing 100mM of either KCl or LiCL The reactions were analysed on denaturing 10% polyacrylamide gels.
